# The Impact of COVID-19 on Emergency Medicine Rotations

**DOI:** 10.7759/cureus.30752

**Published:** 2022-10-27

**Authors:** Robert M Tennill, Matt Turner, Aaron Fleming, Carly Hofreiter, Sharon Kim, Kristin Delfino, Richard Austin

**Affiliations:** 1 Department of Emergency Medicine, Southern Illinois University School of Medicine, Springfield, USA; 2 Department of Center for Clinical Research, Southern Illinois University School of Medicine, Springfield, USA

**Keywords:** clinical competency, covid-19 retro, emergency medicine rotation, covid-19 pandemic, medical education

## Abstract

Introduction

The impact of modifications in curriculum and clinical rotations made secondary to the COVID-19 pandemic on medical education has yet to be fully investigated. We observed differences in the types of patients seen by medical students that may have resulted from clinical disruptions due to the COVID-19 pandemic. We then evaluated what impact these disruptions had on the students’ clinical competency.

Methods

We obtained patient logbooks of third-year medical students (M3) and fourth-year sub-interns (M4) from the first three emergency medicine (EM) rotation blocks of the 2019-2020 (Y19) and 2020-2021 (Y20) academic years. We then reviewed and categorized the chief complaints seen and procedures in which they participated. A robust t-test was used to detect differences in chief complaints and procedures. Finally, we looked for objective differences using the chi-square test in clinical performance between the class of 2021 (Class21) and the class of 2022 (Class22), as assessed by performance on our institution’s clinical competency examination.

Results

Overall, students saw a 25.3% decrease in average number of patient encounters. Statistically significant decreased average numbers of infectious (-28.3%, p=0.013); musculoskeletal (-22.2%, p=0.018); gastrointestinal (GI) (-24.6%, p<0.01); genitourinary (GU) (-33.2%, p<0.01); head, eyes, ears, nose, throat (HEENT) (-31.1%, p<0.01); trauma (-33.0%, p<0.01); and respiratory (-45.4%, p<0.001) complaints were observed.

Both M3s and M4s encountered significantly less GU (-25.6%, p=0.048; -41.7%, p=0.016) and trauma (-29.1%, p=0.023; -33.2%, p=0.032) complaints in Y20. M4s saw significantly less GI complaints (-42.6%, p<0.001) in Y20, whereas M3s encountered significantly less psychiatric and HEENT complaints (-30.3%, p=0.046; -34.6%, p=0.013). Both classes saw significantly less respiratory complaints in Y20 but more so for M4s (-65.3%, p<0.001) than for M3s (-27.9%, p=0.033). There were no significant differences in average number of procedures between years.

We did not observe any differences in overall clinical performance between the two selected classes. While class of 2021 scored a significantly higher average on a case of fatigue (p=0.0004) and class of 2022 on a case of abdominal pain (p<0.0001), there were no significant differences in the primary chief complaints that would be attributed to COVID-19, such as dyspnea.

Conclusion

Modifications made to curricula and clinical rotations due to the COVID-19 pandemic led to students encountering less patients overall, with significant decreases in multiple chief complaint types compared to Y19 but no significant change in procedure numbers. Notably, there was no major impact seen on clinical competency providing a positive argument for considering innovative teaching and learning methods.

## Introduction

The discovery of a novel coronavirus, later termed the severe acute respiratory syndrome coronavirus 2 (SARS-CoV-2), in December 2019 would be the start of the largest pandemic in recent history. As of July 6, 2022, over 500 million cases and over 6.3 million deaths had been recorded worldwide [1].

The first recorded case of COVID-19 in the United States was detected on January 19, 2020, in Washington state [2]. The virus was soon found in several more states, and in just over a month, many states and other governing bodies began enacting various mitigation policies in an attempt to slow this rapid spread [3]. These policies included crowd restrictions, stay-at-home orders, social distancing guidelines, and public face mask mandates, which disrupted many facets of society including education.

On March 17, 2020, the Association of American Medical Colleges (AAMC) took the unprecedented step of strongly supporting medical schools to pause all student clinical rotations in response to the COVID-19 pandemic [4]. National challenges surrounding testing supplies and personal protective equipment likely contributed to this decision, and later, guidance on direct in-person patient care highlighted these two areas as essential for students to return to environments where they may care for COVID-19 patients [5].

While some prior research has looked to determine the potential impact of the COVID-19 pandemic on learning outcomes, the authors could find very little in regard to objective measures of performance in the medical student population specifically those who had been affected by the pandemic. In one such study, Tzeng et al. evaluated medical students in Taiwan in their final year of training through the use of three serial objective structured clinical examinations (OSCEs), comparing those students affected by the pandemic to the prior year’s students that were unaffected. They found that the unaffected group had higher average scores in the final OSCE, as well as an improvement between the second and third OSCEs as compared to the pandemic-impacted group [6]. This was cited as support for the need for supplemental learning opportunities to compensate for lost clinical experiences.

One international survey of 1604 medical student participants from 45 countries found that 81.4% of participants reported an overall negative impact on their training from the COVID-19 pandemic. In addition, a decline in conventional lectures and in-person ward-based teaching was associated with reporting a negative impact on their training, while an increase in clinical responsibilities was associated with less likelihood of reporting a negative impact [7].

An additional study of perceived impact based on 248 medical student responses from various schools across Canada found that 74% reported a reduced quality of education since COVID-19, with 58% of students finding online teaching to be inferior to in-person teaching [8].

Most of the research concerning medical education during the pandemic has focused on how the medical education pedagogy could be adjusted to accommodate the absence of in-person teaching and if these changes could perhaps be implemented as a long-term modification. Lim et al. proposed a decision tree that incorporates the use of substitute educational methods such as online lectures, simulation mannequins, virtual reality environments, videoconferencing, and video-recorded clinical vignettes [9].

In response, various institutions analyzed different learning modalities including initiating a coaching program focused on professional identity and clinical skill development, which showed positive results based on anonymous surveys [10]. Another created a two-week virtual rotation with asynchronous assignments and small group didactic sessions focused on medical knowledge, professional development, professional identity, and social determinants of health again finding positive results based on pre- and post-medical knowledge examinations and surveys [11]. Redinger and Greene created a similar virtual clerkship rotation and found that medical student performance on a national standardized emergency medicine (EM) shelf examination was not significantly different from students who had completed their traditional in-person elective [12]. Nnamani Silva et al. compared the performance of surgical clerkship students who had completed the traditional elective to a pilot virtual course named the surgical extended mastery learning rotation (EMLR), a three-week course created to facilitate learning and eventual resumption of in-person rotations. They found that students enrolled in EMLR showed higher perceived surgical clerkship readiness and had a higher average National Board of Medical Examiners (NBME) surgical shelf examination score than those students enrolled in the traditional pre-COVID-19 elective [13].

While these proposed solutions certainly show promise, the results and perceptions have been mixed; not every medical school has sufficient technological infrastructure to support these alternatives, and there is no agreed-upon standard to ensure quality [14]. Ren et al. surveyed EM clerkship directors and found that while 52% had adapted some form of virtual rotation during the beginning of the pandemic, 71% of these clerkship directors did not feel comfortable completing a standardized letter of evaluation (SLOE) based on the virtual rotation, many citing difficulties evaluating students’ clinical competence [15]. In a systematic review, Kelly et al. confirmed that during the pandemic, many institutions were using online meetings for items such as didactics, laboratory practices, and clinical skill classes but that this might not be ideal for clerkship students as this eliminates the patient-doctor relationship. Ultimately, they concluded that more research is needed to evaluate students’ performance after adapting virtual learning [16].

All medical students at our institution experience a four-week EM core clerkship in their third year. In their fourth year, they have the option to take another four-week EM rotation as a sub-intern for those interested in the specialty as their residency choice. Both rotations include 12 clinical shifts in the emergency department (ED). During the third-year core clerkship, students evaluate patients, construct workups, and participate in and observe procedures as available. The fourth-year rotation includes more responsibility with workups, documentation, and procedures, in addition to participation in simulations, lectures, and skill laboratories.

Following the AAMC’s guidance, our institution paused clinical rotations and in-person lectures in March of 2020 until July 2020. During this time, students were tasked with developing self-study plans along with participating in asynchronous learning sessions, virtual didactics, and virtual simulation and procedure laboratories. Even after their return to clinical environments, students were typically restricted from seeing either COVID-19-positive patients or potential COVID-19 patients. This created significant difficulties for learners, especially in the ED, given the wide variety of presenting COVID-19 symptoms.

While many have pointed out the potential positives of the COVID-19 pandemic on reformatting medical education, few have looked at the actual clinical educational impact on learners. Questions remain over the impact of a national pause on student experiences even after they returned [5]. Others have wondered if there would be curricular gaps created by following the AAMC’s guidance and limiting student exposure to patients in core clerkships [4,5].

Here, we share what impact the COVID-19 pandemic and the responses to the pandemic by academic institutions had on the curriculum of our third-year medical students (M3) and fourth-year medical students. We evaluated if there were differences in the numbers and types of patients and procedures students were exposed to on their EM rotation following their return to the clinical environment after their COVID-19-mandated hiatus (from July to September 2020). We hypothesized that, given the wide variety of symptoms associated with COVID-19, as well as the decreased ED volume, students in clinical rotations during this time period affected by COVID-19 would encounter fewer patients than previous years overall and would see significantly fewer respiratory complaints. Finally, we evaluated whether this decrease in patient encounters, and particularly in respiratory or COVID-19-related chief complaints, led to a meaningful deficit in clinical performance and competency as measured by our institution’s summative clinical competency examination (SCCX).

## Materials and methods

Background

Students from our community-based medical school, with approximately 80 students per class, rotate through the ED at our institution. Our ED is a level-one trauma, ST-segment elevation myocardial infarction (STEMI), and stroke center with approximately 70000 visits per year. During their four-week rotation, students work 12 clinical shifts and keep anonymous records of patient encounters. For each patient encounter, students log the patient’s gender, age, chief complaint, diagnosis, and disposition. They also record procedures they are involved in. Students typically keep both paper and electronic logs of all encounters and submit both at the end of the rotation.

At the end of their third year of medical school at our institution, after they have completed their third-year EM core clerkship, students are required to be evaluated by a summative clinical competency examination (CCX). This consists of 14 standardized patient encounters with varying chief complaints where students are tasked with taking history and performing a physical examination on each patient followed by a computerized assessment of their differential diagnosis, findings, laboratories, problem list, management, diagnosis, and diagnostic justification. Students are also graded on patient satisfaction and a history and physical checklist. Overall, students must pass 10 out of 14 cases with a score of greater than or equal to 65% to successfully pass the summative CCX.

Study design

We obtained the logbooks of third-year medical students (M3) and fourth-year sub-interns (M4) from the first three rotation blocks of the 2019-2020 (Y19) and 2020-2021 (Y20) academic school years (from July to September). We retrospectively reviewed the logbooks and categorized chief complaints seen based on final diagnosis and procedures participated in. We separated the patient chief complaints into the following categories: cardiac; respiratory; gastrointestinal (GI); neurologic; head, eyes, ears, nose, throat (HEENT); musculoskeletal and soft tissue; infectious disease; psychiatric; multisystem trauma; and genitourinary (GU), as well as a miscellaneous category. We separated procedures into the following categories: wound care, bedside ultrasound (BSUS), intubation, fracture reduction, resuscitation, central line, and chest tube, as well as a miscellaneous category. Each encounter was categorized into a single data point, so the total number of encounters is equal to the number of chief complaints documented. We calculated the total and mean numbers of chief complaints and procedures and compared these by class (level of training, M3 versus M4), as well as by academic year. We also compared the chief complaints as a proportion of all complaints seen between academic years. If there were any discrepancies in the number of encounters an individual student logged via paper and online logbooks, the logbook with the largest number of patients was evaluated.

We then obtained the summative CCX scores for the class of 2021 (Class21) and class of 2022 (Class22), where Class21 did not have their third-year EM core clerkship affected by COVID-19 while Class22 was affected. Between the two classes, all 14 chief complaints remained the same; the final diagnoses were also the same with the exception of three cases (case 4, case 6, and case 8 in Table 4). Using group-level analysis, we compared their clinical competency as assessed by their objective performance on the summative CCX. Specifically, we compared their overall mean pass/fail rate and their overall summative CCX mean score and also examined these same data for each of the 14 individual cases. Lastly, we compared the two classes’ objective overall performance on their second-year CCX to ensure there were no significant baseline performance differences between the two classes prior to the COVID-19 pandemic. The institutional review board determined this study as non-human subject research.

Statistical analysis

Continuous variables are described with measures of central tendency and dispersion. Categorical variables are described with frequencies and percents. Differences in the number of chief complains and procedures between groups were assessed with Student’s t-test. Chi-square test and Fisher’s exact test were used to assess the relationship of pass/fail with class year. Significance was determined at the p<0.05 level.

## Results

Total encounters

In Y19, 35 students (22 in M3 and 13 in M4) logged a total of 3680 patient encounters. In Y20, 33 students (18 in M3 and 15 in M4) logged a total of 2592 patient encounters. This translates to an average of 105.14 encounters during the four-week rotation in Y19 and 78.55 in Y20, resulting in a difference of 26.59 or a decrease of 25.3% (p<0-0001). M3s in Y19 logged an average of 111 patient encounters compared to 89 average patient encounters for Y20, a decrease of 19.8% (p<0.01). M4s in Y19 logged an average of 95 patient encounters compared to 66 average patient encounters for Y20, a decrease of 30.8% (p<0.01). Over the first three rotation blocks of the academic year, local ED volumes are estimated to have been down by 14% in Y20 compared to Y19. Nationally, ED volumes decreased by 42% between March and April of 2020, following an increase in volume through July 2020 before stabilizing in August 2020 at levels 15% below the same prepandemic period. It was not until December 2020 when ED volumes again declined to a level 25% lower than the previous year [17].

Chief complaints

Medical students logged one chief complaint for each patient encountered during their rotation. Table 1 displays the average number of chief complaints per student in Y20 compared to Y19. In Y20, they logged a significantly decreased average number for the majority of chief complaints when compared to the average number in Y19. Only cardiac, neurologic, psychiatric, and other complaints did not have statistically significant decreases. When we evaluated the chief complaints as a proportion of all complaints seen in that year, only respiratory chief complaints had a statistically significant decrease of 45.4% from Y19 to Y20 (p<0.0001). Table 2 depicts the breakdown of average chief complaints by year in medical school. For most chief complaints, there were similar trends among M3 and M4 students; however, there were differences in gastrointestinal, HEENT, and psychiatric complaints. M3s saw a significant decline in HEENT and psychiatric complaints (p<0.05), whereas M4s saw a significant decline in gastrointestinal complaints (p<0.001).

**Table 1:** Average encounters for each student by chief complaint Data are reported as mean±SD Y19: 2019-2020 academic year; Y20: 2020-2021 academic year; HEENT: head, eyes, ears, nose, throat

Chief complaints	Y19	Y20	Change	P-value
Cardiac	13.97±4.97	11.58±5.82	-17.1%	0.0720
Gastrointestinal	15.80±5.49	11.91±4.99	-24.6%	0.0033
Genitourinary	5.31±2.19	3.55±2.31	-33.2%	0.0019
HEENT	5.63±2.24	3.88±2.09	-31.1%	0.0014
Infectious	8.29±4.26	5.94±3.16	-28.4%	0.0126
Musculoskeletal	15.23±6.22	11.85±5.16	-22.2%	0.0178
Multisystem trauma	8.77±3.99	5.88±3.52	-33.0%	0.0024
Neurologic	8.97±3.15	7.55±3.23	-15.8%	0.0698
Psychiatric	7.91±4.26	5.94±4.69	-24.9%	0.0724
Respiratory	9.43±3.27	5.15±3.66	-45.4%	<0.0001
Other	5.83±1.95	5.33±3.23	-8.6%	0.4504
Total	105.14±22.68	78.55±28.04	-25.3%	<0.0001

**Table 2:** Average encounters for third-year medical students versus fourth-year medical students by chief complaint Data are reported as mean±SD M3: third-year medical student; M4: fourth-year sub-intern; Y19: 2019-2020 academic year; Y20: 2020-2021 academic year; HEENT: head, eyes, ears, nose, throat *p<0.05

Chief complaints	M3				M4			
	Y19 (n=22)	Y20 (n=18)	Change	P-value	Y19 (n=13)	Y20 (n=15)	Change	P-value
Cardiac	14.41±5.21	12.67±6.23	-12.1%	0.3412	13.23±4.66	10.27±5.19	-22.4%	0.1262
Gastrointestinal	15.18±5.91	13.78±4.62	-9.2%	0.4160	16.85±0.67	9.67±4.59	-42.6%	0.0004*
Genitourinary	5.82±2.30	4.33±2.28	-25.6%	0.0483*	4.46±1.76	2.60±2.03	-41.7%	0.0162*
HEENT	5.86±2.62	3.83±2.23	-34.6%	0.0131*	5.23±1.36	3.93±1.98	-24.9%	0.0576
Infectious	8.95±4.26	6.89±3.23	-23.0%	0.0983	7.15±4.18	4.80±2.76	-32.9%	0.0865
Musculoskeletal	16.41±6.65	12.89±5.41	-21.5%	0.0784	13.23±5.05	10.6±4.72	-19.9%	0.1663
Multisystem trauma	10.18±4.02	7.22±3.84	-29.1%	0.0234*	6.38±2.63	4.27±4.02	-33.1%	0.0319*
Neurologic	9.05±3.46	8.11±2.76	-10.4%	0.3589	8.85±2.67	6.87±3.70	-22.4%	0.1219
Psychiatric	9.73±3.97	6.78±5.09	-30.3%	0.0463*	4.85±2.76	4.93±4.10	-1.7%	0.9487
Respiratory	9.09±2.99	6.56±4.23	-27.8%	0.0327*	10.00±3.76	3.47±1.81	-65.3%	<0.0001*
Other	6.18±1.79	5.89±2.83	-4.7%	0.6923	5.23±2.13	4.67±3.64	-10.7%	0.6280
Total	110.86±23.98	88.94±26.07	-19.8%	0.0087*	95.46±16.99	66.07±25.81	-30.8%	0.0017*

Procedures

Students logged procedures that they performed, assisted in, or observed during their rotation. While students only logged one chief complaint for each patient encountered, they recorded all procedures that they were involved with even if multiple occurred on the same patient encounter. Table 3 displays the average number of procedures students participated in by Y19 and Y20. There were no statistically significant differences in procedures between academic years.

**Table 3:** Average number of procedures participated in by each student during their rotation Data are reported as mean±SD BSUS: bedside ultrasound; Y19: 2019-2020 academic year; Y20: 2020-2021 academic year

Procedures	Y19	Y20	P-value
BSUS	1.83±1.71	1.88±1.78	0.9058
Central line	0.40±0.60	0.61±0.79	0.2289
Chest tube	0.20±0.53	0.24±0.50	0.7364
Fracture reduction	1.49±1.31	2.03±1.49	0.1142
Intubation	1.34±1.37	1.48±1.12	0.6428
Resuscitation	0.71±0.89	0.73±0.88	0.9520
Wound care	3.74±1.87	3.18±2.01	0.2368
Other	4.06±2.93	4.09±2.87	0.9618

Clinical competency

Table 4 displays the summative CCX chief complaints and diagnoses listed, along with a summary of student performance by class. When looking at case 7 (fatigue, obstructive sleep apnea), the average scores were significantly lower for Class22 when compared to Class21 (p<0.001). In case 11 (abdominal pain, peptic ulcer disease), Class22 obtained significantly higher average scores than Class21 (p<0.0001). However, when comparing the overall mean score and average passing rate between the two classes, we did not find any significant differences in their performance (Table 5).

**Table 4:** Third-year summative clinical competency case descriptions, number of pass/fails, and average scores by each case Data are reported as n(%) and mean±SD GAD: general anxiety disorder; HSV: herpes simplex virus; PID: pelvic inflammatory disease; PN: patient notes; NHL: non-Hodgkin’s lymphoma; URI: upper respiratory infection; UTI: urinary tract infection *p<0.05; †Fisher’s exact test used due to small sample size; ‡class of 2021, n=22; class of 2022, n=25; §cases 4, 6, and 8 had different final diagnoses between the classes

Case	Chief complaint	Diagnosis^§^	Class^‡^	Fail	Pass	Chi-square p-value	Average score	P-value
1	Mood changes (PN)	GAD and alcohol abuse	2021	6 (27.3)	16 (72.7)	0.4796^†^	68.18±6.98	0.3862
			2022	4 (16.0)	21 (84.0)		69.96±6.93	
2	Headache	Pseudotumor	2021	6 (27.3)	16 (72.7)	0.7974	68.73±8.45	0.3500
			2022	6 (24.0)	19 (76.0)		71.08±8.59	
3	Fever (PN)	Infected sacral ulcer, UTI, and neglect	2021	7 (31.8)	15 (68.2)	0.5499	69.68±5.73	0.7689
			2022	6 (24.0)	19 (76.0)		69.08±7.89	
4	Vaginal discharge and abdominal pain	PID and domestic violence	2021	2 (9.1)	20 (90.9)	0.4227^†^	74.00±8.34	0.2260
		Primary HSV and domestic violence	2022	5 (20.0)	20 (80.0)		70.72±9.78	
5	Deep breathing and increased thirst	Diabetes mellitus type 1	2021	3 (13.6)	19 (86.4)	0.4703^†^	71.41±8.38	0.5728
			2022	6 (24.0)	19 (76.0)		69.88±9.88	
6	Armpit lump (PN)	NHL	2021	1 (4.6)	21 (95.5)	1^†^	71.82±6.80	0.0713
		Breast cancer	2022	2 (8.0)	23 (92.0)		75.40±6.48	
7	Fatigue (PN)	Obstructive sleep apnea	2021	1 (4.6)	21 (95.5)	0.4681^†^	83.32±7.69	0.0004*
			2022	0 (0.0)	25 (100.0)		75.96±5.37	
8	Dyspnea	Pulmonary embolism	2021	4 (18.2)	18 (81.8)	0.1710^†^	71.64±7.32	0.0502
		COVID-19	2022	1 (4.0)	24 (96.0)		75.36±5.31	
9	Low back pain	Herniated disc	2021	4 (18.2)	18 (81.8)	0.1730	71.32±7.52	0.1628
			2022	9 (36.0)	16 (64.0)		67.68±9.74	
10	Cough	Acute bronchitis and URI	2021	4 (18.2)	18 (81.8)	1^†^	72.36±9.17	0.4189
			2022	4 (16.0)	21 (84.0)		70.40±7.32	
11	Abdominal pain	Peptic ulcer disease	2021	1 (4.6)	21 (95.5)	0.4681^†^	69.64±4.74	<0.0001*
			2022	0 (0.0)	25 (100.0)		77.16±6.24	
12	Edema	Nephrotic syndrome	2021	4 (18.2)	18 (81.8)	1^†^	70.23±8.41	0.8566
			2022	4 (16.0)	21 (84.0)		70.68±8.62	
13	Chest pain	Myocardial infarction	2021	1 (4.6)	21 (95.5)	0.6115^†^	77.45±6.53	0.5682
			2022	3 (12.0)	22 (88.0)		76.24±7.79	
14	Dizziness	Orthostatic hypotension and volume depletion	2021	2 (9.1)	20 (90.9)	0.2529†	73.68±10.64	0.2246
			2022	6 (24.0)	19 (76.0)		70.40±6.90	

**Table 5:** Overall third-year summative clinical competency average scores and percentage passed Data are reported as mean±SD SCCX: summative clinical competency examination; Class22: class of 2022; Class21: class of 2021

SCCX	Class22 (n=25)	Class21 (n=22)	P-value
Average scores	72.16±4.33	72.36±4.27	0.8721
Percentage passed	84.08±13.49	85.18±17.99	0.8119

In order to account for any baseline class differences prior to the COVID-19 pandemic, we also compared the objective overall performance of both classes’ second-year CCX scores and did not find any significant differences (Table 6).

**Table 6:** Overall second-year clinical competency average scores and percentage passed Data are reported as mean±SD Y2 CCX: second-year clinical competency examination; Class22: class of 2022; Class21: class of 2021

Y2 CCX	Class22 (n=24)	Class21 (n=22)	P-value
Average scores	74.01±3.59	72.51±4.20	0.1998
Percentage passed	88.90±12.80	83.00±12.80	0.1248

## Discussion

Overall

While the 14% decrease in ED patient volume at our institution during the study comparison most likely contributed to the decreased number of patient encounters, perhaps more impact was felt by limiting the types of patient encounters. Unfortunately, we are unable to account for whether the 14% decrease in volume over the time period also had a significantly different breakdown of chief complaints in Y20 compared to Y19 that might have impacted the types of patients presenting to the ED for the students to evaluate. However, restricting students from evaluating potentially COVID-19-related chief complaints likely played a significant role in further reducing overall patient encounters, and this was likely the practice done nationally as well.

Clinical encounters and chief complaints

Students had a statistically significant decrease in the number of patient encounters for almost every chief complaint. As might be suggested, attempting to limit student exposure likely decreased the number of respiratory chief complaints they saw on their rotation in particular. Respiratory chief complaints were nearly 50% down compared to previous years for all students and specifically for M4s as they saw 65% fewer respiratory patients. In other words, a sub-intern would typically expect to work through the differential diagnosis, workup, management, and disposition on 10 patients with respiratory symptoms during their rotation; for Y20, they did so on less than four patients. While the authors are unaware of any published data to suggest that there is a minimum number of times a student must encounter a particular chief complaint to be proficient at evaluating that chief complaint, it does appear that students training during the pandemic limitation period had a different experience than the groups before them. When we evaluated the chief complaints as a proportion of all complaints seen in that year, only respiratory chief complaints had a statistically significant decrease from Y19 to Y20, which is both expected given their instruction to avoid those chief complaints but also suggestive of an area of potential knowledge deficit. Given that respiratory complaints typically account for approximately 10% of ED visits, the second component of our study focused on whether this led to any deficits in clinical competency, which might indicate an area graduate medical education programs need to focus on early [18].

Some of the significant decreases can be attributed more to one class than the other. For psychiatric complaints, M3s saw a statistically significant decrease, whereas M4s did not. This is likely in part due to the lower number of psychiatric patients seen by M4s in Y19 compared to M3s. Our anecdotal experience is that M4s tend to see less psychiatric chief complaints when they are expected to have a larger role in the primary management of their patients as these chief complaints tend to have longer length of stays in our EDs and their care is more frequently transitioned to an oncoming provider instead of obtaining a final disposition.

For GI chief complaints, we note that M4s had a more significant decrease in those encounters compared to M3s. We did not study or attempt to ascertain why this difference exists. It is possible that M4s, given an extra year of training and clinical knowledge, more closely associated GI symptoms with COVID-19 than M3s did and were more likely to avoid that population.

While all chief complaints were affected, it is reassuring that some common chief complaints, including chest pain and neurologic and psychiatric emergencies, were still seen in numbers that were not statistically significant and likely a reflection of the overall decrease in ED volume.

Procedures

There were no significant differences in numbers of procedures that learners participated in between Y19 and Y20. This was slightly unexpected, as we had anticipated a decrease in the number of intubations since students were to avoid respiratory chief complaints, and based on our data, they did avoid those patients.

It is possible that overall procedural exposure was less affected as the significant decrease in patient volume allowed more time for students to engage with other areas of the department where procedures were taking place. In addition, students may have been more likely to be encouraged to get involved with procedures occurring throughout the department such as trauma airways given the increase in downtime on shift due to decreased patient volumes. It may have also been easier for students to get involved with observing procedures while still following protocol by standing outside the room at a distance or behind a glass door. While there is clearly an educational difference between performing a procedure and watching from a distance, this strategy would allow them to feel that they observed the procedure, but they likely didn’t feel that they participated in the differential, workup, and disposition of the patient. This may have led to logging the experience as a procedure but not as a completed clinical encounter.

Impact on clinical competency

While the decrease in overall clinical patient encounters and particularly respiratory complaints was evident, the essential question for undergraduate and graduate medical education programs was whether this would lead to deficits in clinical competency. Fortunately, the results of this single-institution study suggest that overall clinical competency was not affected by this decrease in patient encounters due to the COVID-19 pandemic. This held true even when considering clinical competency related to respiratory chief complaints. In addition, this may speak to the importance and success of the innovative teaching and learning methods that were used throughout clinical restrictions brought on by the pandemic. Strategies such as moving to an online learning platform, distanced learning, and virtual simulation and procedural sessions may have contributed successfully to preventing these clinical competency deficits. Moreover, while it is outside of the scope of this study, it may provide a potential argument for competency-based medical education (CBME). Perhaps, fewer patient encounters are necessary to develop clinical competency, and this curricular time could be better spent elsewhere with alternative teaching and learning modalities. Further exploration is warranted.

Limitations

While many students log these encounters during a shift, some do so after shifts as the logs are not collected until the end of the rotation. This leads to potential student recall bias affecting data collection; however, the logging process did not change between Y19 and Y20. Furthermore, our rotations do not have a minimum expectation of number of patients evaluated, and there was a wide range of numbers of patients logged in both years. It is possible that students underreported the number of patient encounters they had, as there is no formal evaluation of the numbers that they choose to log.

One unexpected difficulty was a student who was logging patients that they did not personally encounter but rather discussed with a resident or an attending. The student did make note of this in their logbook. This was particularly noted with respiratory complaints, which students were discouraged from seeing. Discussions such as these would not typically be logged. These specific examples with this student were not counted in the formal analysis, but it is possible that other students logging similar discussions as encounters may have occurred without this acknowledgment, and inadvertently, this was included in the formal data analysis. 

Regarding the study of individual chief complaints, we note the decrease in our ED volume over the period of comparison but do not know the breakdown of chief complaints over that period. It is possible that the pandemic had an effect on the types of chief complaints that presented and may explain why some chief complaints were seen less than anticipated based just off of a 14% decrease in volume. However, regardless of the cause of the decreased numbers, be it due to factors related to COVID-19, curriculum restrictions, or likely a combination of both, the data suggest that our medical students during this time period encountered less patients across the spectrum of chief complaints than their peers in the previous year.

A potential confounder of note is the complicated relationship between clinical learning on the emergency medicine clerkship rotation and future performance on our institution’s summative CCX following the completion of all clerkship rotations. This particular examination was chosen as our measure of clinical competency as it is the closest in proximity to the students’ EM clerkship and the only objectively scored competency examination that is taken by all third-year students at our institution. While performance on the summative CCX is most certainly influenced by the students’ experience on all core clerkships, all clerkships at our institution were instructed to use the same restrictions on evaluating possible COVID-19 patients throughout the academic year, and alternatively, adjunctive teaching and learning modalities were commonplace.

Finally, this study was looking at patient encounter data only over the course of a three-month period in consecutive years at our single institution. As this was a single-center study, results may not be generalizable to other centers. However, this time period did directly compare pre-COVID-19 patient volumes to a corresponding peak of our patient volume decline secondary to the pandemic.

## Conclusions

The COVID-19 pandemic caused major changes in almost all aspects of life, and medical education was not exempt. Actions made to protect our learners, as well as changes in the makeup of the ED patient population, resulted in a different clerkship and sub-intern experience for learners during the first three blocks of the 2020-2021 academic year. Here, we give objective data showing a decrease in the number of patient encounters and how student exposure to various chief complaints was impacted. Despite the decreases in the number of patient encounters, the procedural experience as captured by logging appeared unchanged.

Encouragingly, while clinical patient encounters were clearly reduced during this time period affected by the pandemic, this did not lead to a meaningful difference in clinical competency as measured by our institution’s summative CCX. This may provide a positive argument for the success of alternative and innovative teaching and learning modalities. Strategies such as self-study plans with asynchronous learning sessions, online learning platforms, distanced learning, virtual didactics, and virtual simulation and procedure laboratories, as employed by our institution and many others, appear to have had a positive impact. Many of these modalities are likely to persist at some level and may be incorporated for a more flexible and dynamic curriculum. At a minimum, this study suggests that these strategies may be a useful adjunct should a similar situation with limits to clinical learning arise. More work needs to be done to determine whether these findings can be extrapolated to additional educational institutions, in addition to which alternative teaching and learning modalities are most beneficial.
